# Revamping Non-Small Cell Lung Cancer Treatments in Stages II and III: Preparing Healthcare for Cutting-Edge Immuno-Oncology Regimens

**DOI:** 10.3390/cancers16162842

**Published:** 2024-08-14

**Authors:** Luca Bertolaccini, Monica Casiraghi, Claudia Bardoni, Cristina Diotti, Matteo Chiari, Antonio Mazzella, Filippo de Marinis, Lorenzo Spaggiari

**Affiliations:** 1 Department of Thoracic Surgery, IEO European Institute of Oncology, IRCCS, Via Ripamonti 435, 20141 Milan, Italy; monica.casiraghi@ieo.it (M.C.); claudia.bardoni@ieo.it (C.B.); cristina.diotti@ieo.it (C.D.); matteo.chiari@ieo.it (M.C.); antonio.mazzella@ieo.it (A.M.); lorenzo.spaggiari@ieo.it (L.S.); 2 Department of Thoracic Oncology, IEO European Institute of Oncology, IRCCS, 20141 Milan, Italy; 3 Department of Oncology and Hemato-Oncology, University of Milan, 20122 Milan, Italy; filippo.demarinis@ieo.it

**Keywords:** lung cancer, management, neoadjuvant treatments

## Abstract

**Simple Summary:**

Non-small cell lung cancer in stages II and III is a significant health challenge, requiring ongoing improvements in treatment strategies. One promising approach is neoadjuvant therapy, which is given before surgery to shrink tumours and treat unseen cancer spread. This early treatment can help doctors see how well the tumour responds, which can guide further therapy after surgery. Testing for specific markers in the tumour, like certain gene mutations and protein levels, helps doctors choose the most effective treatments for each patient. By doing this, the chances of successful surgery and long-term survival improve. Managing this type of cancer requires a team of specialists working together to create personalised treatment plans. Despite its promise, neoadjuvant therapy comes with challenges, such as determining which tumours can be removed after treatment and recognising if a tumour appears larger due to treatment effects rather than actual growth. However, the medical community’s ongoing research and dedication are essential to overcoming these obstacles and enhancing the benefits of neoadjuvant therapy. This approach aims to increase survival rates and improve the quality of life for patients with non-small cell lung cancer, making it a valuable advancement for society.

**Abstract:**

Non-small cell lung cancer (NSCLC) poses a significant challenge in clinical oncology, necessitating continual refinement of treatment approaches in stages II and III. Recent advancements have highlighted the potential of neoadjuvant therapy in optimising patient outcomes. Biomarker testing guides neoadjuvant therapy decisions, including epidermal growth factor receptor (EGFR) mutation and programmed death-ligand 1 (PD-L1) expression testing. Neoadjuvant therapy aims to improve oncological outcomes by treating micrometastatic disease and assessing tumour response before surgery. Disease-free survival is a surrogate endpoint for overall survival in both neoadjuvant and adjuvant settings. Multidisciplinary collaboration is crucial for individualised treatment planning and optimising patient care. The management of NSCLC requires a comprehensive approach, integrating expertise across disciplines and tailoring treatment strategies to individual patient needs. Neoadjuvant therapy shows promise in improving long-term outcomes, with biomarker testing guiding treatment decisions. Challenges such as defining borderline resectability and differentiating pseudoprogression highlight the need for ongoing research and collaboration.

## 1. Introduction

The management of non-small cell lung cancer (NSCLC) presents a complex and multifaceted challenge for oncologists and thoracic surgeons alike [1]. In recent years, significant strides have been made in understanding the intricacies of NSCLC biology, leading to the development and refinement of novel therapeutic strategies to improve patient outcomes. In the current landscape of clinical oncology, the management of NSCLC has witnessed a notable shift, prompting a reassessment of treatment approaches. Traditionally, adjuvant chemotherapy, typically a platinum-based doublet administered for 4–6 cycles, has been a standard for patients with stage II and III NSCLC. The emergence of immunotherapy, particularly with immune checkpoint inhibitors (ICIs), has further complicated the decision-making process. A noteworthy trend in clinical practice is the exploration of neoadjuvant approaches [2]. The impact of ICIs has been revolutionary, showcasing durable disease control in selected patients when administered either as standalone treatments or in combination with other therapeutic modalities. These inhibitors have demonstrated notable advancements in OS and redefined the treatment standards in both locally advanced and metastatic settings [3]. One such strategy that has garnered increasing attention and investigation is utilising neoadjuvant therapy in the management of NSCLC. Neoadjuvant therapy, administered before surgical resection, promises to optimise tumour control, tailor subsequent treatments, and potentially improve long-term survival. However, navigating the complexities of neoadjuvant therapy requires careful consideration of numerous factors, including patient selection, treatment regimens, and endpoints for assessing treatment efficacy. In this context, a thorough understanding of the role of neoadjuvant therapy in NSCLC becomes paramount. From baseline staging investigations to postoperative surveillance, each step in the treatment journey presents unique challenges and opportunities for optimising patient care. Key considerations include selecting appropriate biomarker testing to guide treatment decisions, assessing tumour response to neoadjuvant therapy, and correlating surrogate endpoints such as disease-free survival (DFS). However, the most crucial factor in this journey is the role of multidisciplinary collaboration. This collaboration among oncologists, thoracic surgeons, radiologists, and pathologists is essential; it is the backbone of formulating individualised treatment plans, interpreting imaging and pathological findings, and coordinating seamless transitions between preoperative and postoperative care It is this collaboration that ensures the success of neoadjuvant treatment in NSCLC, and it is this collaboration that we must continue to foster and strengthen [2].

### Diagnosis and Preoperative Assessment

Several baseline staging investigations are typically included to guide treatment decisions. These investigations aim to assess the extent of disease involvement accurately, determine the feasibility of curative therapy, and inform treatment planning. The specific staging investigations should include a chest and abdomen computed tomography (CT) scan, positron emission tomography (PET) scan, brain imaging (magnetic resonance imaging or contrast-enhanced CT scan), pulmonary function tests, accurate mediastinal staging with endobronchial ultrasound-guided/endoscopic ultrasonography transbronchial needle aspiration (EBUS/EUS TBNA), or endoscopic ultrasound-guided fine-needle aspiration. The molecular profiling of tumour tissue for actionable genetic mutations is increasingly essential in guiding treatment decisions, particularly for targeted therapies and immunotherapy, and should be decided by the medical oncologist (Table 1) [4,5].

Treatment decisions should be reviewed at several critical points throughout the disease trajectory to ensure optimal management and address changing clinical circumstances. The timing of treatment decision reviews may vary based on disease stage, treatment modality, response to therapy, and the emergence of new clinical information. A multidisciplinary team (MDT) of specialists should discuss each newly diagnosed NSCLC case. Before initiating treatment, treatment decisions should be reviewed with the patient to obtain informed consent, discuss potential benefits and risks, and address any concerns or preferences. Periodic assessment of treatment response, typically through a follow-up CT scan, should prompt a review of treatment. An MDT comprises specialists from various disciplines, including medical oncology, radiation oncology, thoracic surgery, pulmonology, radiology, pathology, and supportive care. Each member brings unique expertise and perspectives, facilitating a comprehensive evaluation of the patient’s disease status, treatment options, and overall care needs. MDT discussions facilitate evidence-based decision-making, weighing each treatment option’s benefits, risks, and potential outcomes in the patient’s clinical situation. MDT discussions promote consensus building, shared decision-making, and alignment of treatment goals across disciplines, ultimately enhancing the quality and effectiveness of patient care [1,6].

## 2. Adjuvant and Neoadjuvant Systemic Therapies

Biomarker testing plays a crucial role in guiding neoadjuvant therapy for NSCLC patients, helping to identify molecular alterations that can inform treatment decisions and predict treatment response. Epidermal growth factor receptor (EGFR) mutation testing is recommended with adenocarcinoma histology. EGFR mutations predict response to EGFR tyrosine kinase inhibitors (TKIs) such as gefitinib, erlotinib, or osimertinib. Testing for anaplastic lymphoma kinase (ALK) and ROS1 rearrangements is recommended in adenocarcinoma (Table 2). Detection of ALK or ROS1 rearrangements indicates potential responsiveness to ALK inhibitors (e.g., crizotinib, ceritinib) or ROS1 inhibitors (e.g., crizotinib, entrectinib), respectively [7].

Adjuvant treatment options for stage II and III NSCLC have increasingly included targeted therapies for patients with specific genetic mutations. The FDA and EMA have approved regimens based on significant clinical trial evidence. The ADAURA trial is pivotal in demonstrating the efficacy of osimertinib, a third-generation EGFR TKI, as an adjuvant treatment. This study showed a substantial improvement in DFS for patients with resected stage IB–IIIA EGFR-mutated NSCLC. The trial reported a median DFS not reached for the osimertinib group compared to 19.6 months for the placebo group, yielding a hazard ratio of 0.17 (*p* <0.0001) [8].

Consequently, the FDA approved osimertinib for this indication on December 18, 2020, recognising its effectiveness irrespective of prior adjuvant chemotherapy [9]. Other studies, such as ADJUVANT-CTONG1104, have compared gefitinib to standard chemotherapy (vinorelbine plus cisplatin) in a similar patient population, further supporting the role of EGFR TKIs in the adjuvant setting [9]. For patients with ALK-positive NSCLC, the ALINA trial evaluated alectinib, an ALK inhibitor, versus observation in patients with resected stage IB–IIIA disease. Alectinib is associated with a clinically meaningful benefit concerning CNS disease-free survival compared to chemotherapy (hazard ratio for CNS disease recurrence or death = 0.22; 95% CI: 0.08 to 0.58) [10]. Consequently, the FDA approved osimertinib for this indication on April 18, 2024, recognising its effectiveness irrespective of prior adjuvant chemotherapy.

Programmed death-ligand 1 (PD-L1) expression testing is recommended for all NSCLC patients, particularly those considered for immunotherapy. PD-L1 expression levels assessed by immunohistochemistry help predict response to ICIs, such as pembrolizumab, nivolumab, or atezolizumab.

Neoadjuvant immunotherapy with ICIs may be considered for patients with high PD-L1 expression levels, aiming to induce tumour regression and enhance operability before surgery [7]. Assessing the stage of the NSCLC tumour is essential to determine its extent of spread and resectability. Neoadjuvant therapy is typically considered for patients with locally advanced (stage II–III) NSCLC, where downstaging may increase the likelihood of successful surgical resection. Larger tumours or those involving adjacent structures may pose challenges for complete resection and may require multimodal therapy. Most relevant for determining the operability of patients are pulmonary function tests and, when in doubt, bicycle ergometry. Assessing the patient’s performance status using tools such as the Eastern Cooperative Oncology Group (ECOG) performance status scale may also help determine their ability to tolerate surgery and subsequent treatment. Evaluating pulmonary and cardiac function helps identify preexisting conditions that may impact surgical candidacy or perioperative management. Considering psychosocial factors, including social support, mental health status, and patient preferences, helps assess the patient’s ability to cope with the challenges of surgery and postoperative recovery. Engaging in shared decision-making discussions with patients is essential to understand their treatment preferences, values, and care goals. Patients should have a clear understanding of the risks, benefits, and alternatives to neoadjuvant therapy and surgery. Neoadjuvant therapy allows a more personalised treatment approach by tailoring the systemic therapy regimen based on individual patient factors, tumour characteristics, and biomarker characteristics. In neoadjuvant treatment for NSCLC, DFS serves as an essential surrogate endpoint for OS but may not always perfectly correlate with OS. DFS measures the time from initiating treatment (neoadjuvant therapy) to the recurrence of disease or death from any cause.

Durvalumab has emerged as the standard adjuvant treatment following chemoradiotherapy for patients with stage III unresectable NSCLC. This treatment strategy is supported by findings from the PACIFIC trial, which demonstrated that durvalumab significantly improves DFS and OS compared to placebo in patients who have not experienced disease progression after concurrent chemoradiotherapy. Specifically, the median DFS was notably longer in patients receiving durvalumab, highlighting its efficacy as a consolidation therapy. Furthermore, durvalumab’s safety profile was comparable to that of placebo, with manageable side effects, making it a viable option for enhancing patient outcomes in this challenging cancer stage [11].

A prolonged DFS indicates a successful treatment response, effectively controlling the primary tumour and micrometastatic disease. As such, a favourable DFS is often associated with improved OS, as patients who remain disease-free longer are more likely to survive longer. DFS is a surrogate marker for long-term outcomes, including OS, in neoadjuvant therapy trials. While DFS is a valuable endpoint in neoadjuvant therapy trials, it may only partially capture the impact of treatment on OS, especially in the context of long-term follow-up. In adjuvant therapy for NSCLC, DFS serves as a surrogate endpoint for OS [12]. The correlation between DFS and OS in the context of adjuvant therapy reflects the impact of treatment on delaying disease recurrence or progression, which ultimately influences long-term survival outcomes. A longer DFS following adjuvant therapy is associated with improved OS outcomes, reflecting the treatment’s effectiveness in delaying disease recurrence, controlling disease progression, and ultimately prolonging patient survival (Table 3) [13].

## 3. Discussion

In 1995, a pivotal meta-analysis by the Non-Small Cell Lung Cancer Collaborative Group revealed a 5% OS benefit at five years, particularly for patients with stage II and IIIA disease [14]. Subsequently, numerous randomised clinical trials were conducted to explore the efficacy of adjuvant chemotherapy in resected early-stage NSCLC. One noteworthy meta-analysis by the LACE Collaborative Group demonstrated a 5.4% reduction in the risk of death at five years with adjuvant chemotherapy compared to no chemotherapy [15]. They were shifting the focus to neoadjuvant chemotherapy for locally advanced NSCLC; historical therapeutic strategies for stage III disease involved radiation therapy, which showed poor survival outcomes [16].

The debate on neoadjuvant vs. adjuvant immunotherapy in early-stage NSCLC involves weighing the advantages and disadvantages of each approach. The choice between neoadjuvant and adjuvant therapy may depend on individual patient characteristics and the evolving landscape of immunotherapy and targeted treatments [17]. The debate between neoadjuvant and adjuvant immunotherapy in treating early-stage NSCLC encompasses several factors and considerations. Neoadjuvant immunotherapy offers distinct advantages, such as the early eradication of micrometastases, a higher treatment initiation rate, increased patient adherence, potential surgical downstaging, and the ability to assess pathologic responses, which can serve as predictors of survival. In addition, adjuvant immunotherapy allows for the fastest time to surgery, eliminating risks of complications from systemic therapy before surgery. It also provides a more extended treatment duration for systemic control, and the flexibility of post-surgery time allows patients more time to recover (Table 4).

Recent clinical trials, such as AEGEAN [18], KEYNOTE-671 [19], CheckMate77T [20], and IMpower030 [21], explore neoadjuvant and adjuvant treatment approaches, including chemoimmunotherapy (Table 5). These trials aim to advance our understanding of the optimal treatment strategies for early-stage NSCLC. The debate on neoadjuvant chemotherapy in NSCLC focuses on improving outcomes for patients diagnosed at advanced stages. Prognostic factors and clinical outcomes after neoadjuvant therapy in the immunotherapy era highlight the importance of complete surgical resection, tumour downstaging, and achieving a major pathological response (MPR) with 10% or less residual tumour [17].

The exploration of neoadjuvant ICIs in the early-stage setting, exemplified by the phase II CheckMate 159 trial, has provided valuable insights into this approach’s feasibility, efficacy, and safety for resectable stage IB–IIIA NSCLC [2]. The 5-year relapse-free survival and OS rates were reported at 60% and 80%, respectively, further emphasising the promising outcomes of this neoadjuvant ICI strategy. Neoadjuvant ICIs demonstrated generally manageable safety profiles. Curative surgery was performed in almost 90% of patients across most trials [3].

Any surgical approach in NSCLC aims to achieve complete resection (R0) according to well-defined criteria associated with superior OS. Adjuvant therapy, while advantageous in not delaying surgical resection, has limitations as its benefits depend on the stage at diagnosis. Conversely, neoadjuvant treatment offers several advantages, including creating an optimal environment for immune response development, preserving intact vasculature around the tumour bed, and enhancing treatment compliance. Moreover, evaluating clinical and/or pathological responses improves patient selection and prognostication. The neoadjuvant approach also provides a unique opportunity to investigate potential biomarkers of ICI efficacy in both initial biopsy-derived and surgical specimens. However, neoadjuvant strategies come with inherent risks, such as possible delays in surgical resection, induction therapy-related morbidity or toxicities leading to the cancellation of surgery, and an increased risk of intraoperative technical difficulties compared to patients not receiving neoadjuvant therapy. In trials testing perioperative strategies, up to 20% of patients did not undergo surgery, with progressive disease precluding surgery reported in only 5–10% of cases. Thoracic surgeons should be prepared for potential challenges, such as difficult dissection of pulmonary artery branches due to inflammatory reactions induced by neoadjuvant therapy [3].

OS remains the paramount endpoint in clinical trials involving patients with early-stage NSCLC. However, its utilisation in such trials poses challenges due to the extended median time required to reach this endpoint compared to advanced-stage disease. The complexity is further compounded by adopting ICIs as the standard of care for metastatic disease. Surrogate endpoints, particularly MPR and pathological complete response (pCR), have been incorporated into neoadjuvant trials to address these challenges. Historically, pCR has demonstrated a robust correlation with OS in neoadjuvant chemotherapy trials. MPR and pCR have been adopted as co-primary endpoints in most neoadjuvant ICI trials, with pCR also showing potential as a surrogate marker for DFS and OS in specific trials. However, definitive evidence of pCR serving as a surrogate for OS in NSCLC still needs to be improved, necessitating longer follow-up data for confirmation. The definition and reproducibility of MPR remain subjects of ongoing debate, posing challenges to its potential association with OS. Future advancements, such as using artificial intelligence platforms, could enhance the determination of MPR, improving its validity as a surrogate endpoint in neoadjuvant (chemo)immunotherapy trials [3]. Recent trials, including NeoCOAST, have adopted neoadjuvant platform designs incorporating novel surrogate endpoints. These endpoints aim to facilitate rapid data generation to inform subsequent trials evaluating new immunotherapy-based combination regimens for early-stage resectable NSCLC. Immunotherapy may also offer an unexpected benefit in preventing secondary malignancies [23].

Adjuvant therapy also facilitates molecular testing during the recovery phase, enabling the exploration of molecular alterations with adequate specimens to guide the selection of subsequent adjuvant therapy. Adjuvant therapy distinguishes itself from neoadjuvant treatment with ICIs by obviating the need for rapid molecular determination. The significance of achieving a pCR remains to be fully realised, with adjuvant therapy potentially offering diminished value in patients who achieve pCR. Circulating tumour DNA, commonly used for molecular driver detection in metastatic NSCLC, faces uncertainty regarding its application in detecting minimal residual disease after resection and guiding adjuvant therapy, primarily due to sensitivity challenges in the earlier stages of the disease. The choice between neoadjuvant and adjuvant immunotherapy hinges on weighing these benefits and considerations, considering factors such as treatment initiation rate, surgical downstaging potential, and the impact on molecular testing and surveillance [24].

A delay in initiating definitive treatment correlates with increased cancer-associated mortality. This delay may stem from the absence of a formal definition of borderline resectability, a term specific to individual surgeons and centres, encompassing patients for whom achieving a ‘complete resection’ may be challenging. Therapeutic discussions and decisions are rapidly evolving, with emerging data indicating the safety and efficacy of adding novel therapies, such as immunotherapy, in the neoadjuvant setting [25].

Restaging disease after neoadjuvant therapy is a critical step in the treatment pathway, with both contrast-infused CT and PET/CT providing sufficient radiographic information. However, early reports following neoadjuvant immuno-chemotherapy have identified instances where patients exhibited changes suggestive of radiological progression posttreatment, not substantiated by pathological findings. This phenomenon, termed pseudoprogression, involves an inflammatory rather than infiltrative process characterised by increases in the size or avidity of the primary lesion or adjacent lymph nodes. Although not uncommon, the incidence of pseudoprogression varies among reports. Differentiating pseudoprogression from true progression or hyperprogression is crucial, as pseudoprogression is associated with improved disease survival, while hyperprogression leads to more dismal outcomes. However, delaying treatment while additional investigations are conducted could also negatively impact survival outcomes. Therefore, suspected progression during restaging necessitates cautious interpretation of PET/CT and CT imaging results, with the urgent exclusion of progressive disease to determine the most appropriate therapy. Limited access to repeat PET/CT may preclude its use for restaging, but its absence may not significantly affect the incidence of missed disease progression.

## 4. Conclusions

The management of NSCLC requires a multidisciplinary approach and careful consideration of evolving treatment strategies. Neoadjuvant therapy shows promise in optimising tumour control and improving long-term outcomes, while biomarker testing guides personalised treatment decisions. Challenges such as defining borderline resectability and differentiating pseudoprogression underscore the importance of ongoing research and collaboration. By integrating expertise, individualising treatment plans, and prioritising patient-centred care, healthcare teams can strive to optimise NSCLC management and enhance patient outcomes within the healthcare system.

## Figures and Tables

**Table 1:** Baseline Staging Investigations for non-small cell lung cancer (NSCLC). CT = Computed tomography; EBUS TBNA = Endoscopic ultrasound-guided transbronchial fine-needle aspiration; MRI = Magnetic resonance imaging; PET = Positron emission tomography.

Investigation	Purpose	Details
**Chest and abdomen CT scan**	Assess primary and metastatic disease spread	Provides detailed images of the chest and abdomen to detect primary tumours and metastases
**PET scan**	Evaluate metabolic activity of lesions	Detects areas of increased metabolic activity, helping to identify malignant lesions and metastases
**Brain imaging (MRI or contrast-enhanced CT scan)**	Detect brain metastases	MRI or CT scans of the brain to identify the presence of metastatic lesions in the brain
**Pulmonary function tests (FEV1, DLCO, and ergometry)**	Assess respiratory function	Evaluates lung function to determine the patient’s ability to tolerate surgery and other treatments
**Endobronchial ultrasound-guided transbronchial needle aspiration**	Accurate mediastinal staging	Provides tissue samples from mediastinal lymph nodes to assess for metastatic involvement
**Endoscopic ultrasound-guided fine-needle aspiration**	Accurate mediastinal staging	Complements EBUS TBNA by providing additional tissue samples from lymph nodes for staging purposes
**Molecular profiling of tumour tissue**	Guide targeted and immunotherapy treatments	Identifies actionable genetic mutations and biomarkers, informing decisions for targeted therapies and immunotherapy

**Table 2:** Biomarker Testing and Associated Therapies in non-small cell lung cancer.

Biomarker	Histology	Associated Therapies	Details
**EGFR mutation**	Adenocarcinoma	EGFR TKIs (e.g., gefitinib, erlotinib, osimertinib)	Predicts response to EGFR tyrosine kinase inhibitors
**ALK rearrangement**	Adenocarcinoma	ALK inhibitors (e.g., crizotinib, ceritinib)	Indicates potential responsiveness to ALK inhibitors
**ROS1 rearrangement**	Adenocarcinoma	ROS1 inhibitors (e.g., crizotinib, entrectinib)	Like ALK rearrangements, guides use of ROS1 inhibitors for targeted treatment
**PD-L1 expression**	All NSCLC	ICIs (e.g., pembrolizumab, nivolumab, atezolizumab)	High PD-L1 expression predicts response to immune checkpoint inhibitors, guiding neoadjuvant immunotherapy decisions

**Table 3:** Comparison of Neoadjuvant and Adjuvant Therapies in Non-small Cell Lung Cancer. DFS = Disease-Free Survival; MPR = Major Pathological Response; pCR = Pathological Complete Response.

Aspect	Neoadjuvant Therapy	Adjuvant Therapy
**Timing**	Administered before surgical resection	Administered after surgical resection
**Primary Goals**	Reduces tumour size, improves operability, and eliminates micrometastases	Eliminate residual disease, prevent recurrence, and improve survival
**Benefits**	Early eradication of micrometastases Potential for tumour downstaging, increasing the likelihood of complete resection Pathological response assessment provides prognostic information Intact vasculature around the tumour bed can enhance treatment efficacy	Immediate surgical intervention without delay caused by preoperative treatments Prolonged treatment duration for systemic control Allows for molecular testing during recovery to guide subsequent therapy decisions Avoids complications and toxicities associated with preoperative systemic therapy
**Challenges**	Potential delays in surgery due to treatment-related toxicities or disease progression Risk of increased perioperative morbidity and technical difficulties during surgery	Benefits may vary depending on the stage at diagnosis and residual disease postsurgery May miss the opportunity for downstaging that could improve surgical outcomes
**Surrogate Endpoints**	MPR and pCR can be early indicators of long-term outcomes, but definitive correlation with OS is still under investigation	DFS serves as a surrogate for OS, indicating the treatment’s effectiveness in preventing recurrence and prolonging survival
**Patient Selection**	Typically considered for locally advanced (stage II–IIIA) NSCLC Requires careful evaluation of performance status, pulmonary and cardiac function, and psychosocial factors	Suitable for patients postsurgery to target residual disease Depends on surgical outcomes and pathological findings postresection

**Table 4:** Comprehensive overview of current immuno-oncological treatment modalities, highlighting their mechanisms and roles in cancer therapy.

Treatment Modality	Mechanism of Action	Role in Cancer Treatment
**Immune Checkpoint Inhibitors (ICIs)**	Blockade of immune checkpoints (e.g., PD-1, CTLA-4) to enhance T cell activation and proliferation	Enhances the immune response against tumours
**Chimeric Antigen Receptor (CAR) T Cell Therapy**	T cells are genetically modified to express CARs that target specific tumour antigens	Directly kills cancer cells
**Cancer Vaccines**	Stimulates the immune system to recognise and attack cancer cells	Prevents cancer recurrence and treats existing tumours
**Oncolytic Virus Therapy**	Uses genetically modified viruses to selectively infect and kill cancer cells	Induces systemic anti-tumour immunity
**Dendritic Cell Therapy**	Dendritic cells are activated and used to present tumour antigens to T cells	Enhances T cell response to cancer
**Combination Immunotherapy**	Combines multiple modalities to enhance therapeutic efficacy	Synergistic effects to improve response rates
**Cytokine Therapy**	Administration of cytokines (e.g., IL-2, IFN-α) to boost immune response	Enhances immune cell activity

**Table 5:** Surgical outcomes in neoadjuvant and perioperative trials for non-small cell lung cancer. Adapted from [22]. MPR = Major Pathological Response, PCR = Pathological Complete Response, R0 = Complete Resection with No Residual Tumour. N/A = not applicable.

Trial	Intervention	MPR Rate (%)	PCR Rate (%)	Surgical Resection Rate (%)	Minimally Invasive Surgery Rate (%)	R0 Resection Rate (%)	Control Group MPR Rate (%)	Enrolled Patients’ Stages
**LCMC3**	Neoadjuvant atezolizumab	20	6	88	54	91	10	Stages II–III
**NEOSTAR**	Neoadjuvant nivolumab	22	9	91	29	100	12	Stages IIIA–B
**NEOSTAR**	Neoadjuvant nivolumab + ipilimumab	38	29	76	26	100	15	Stages IIIA–B
**KEYNOTE-671**	Neoadjuvant pembrolizumab + chemo	30.2	18.1	82.1	N/A	92	11	Stages II–III
**CHECKMATE-816**	Neoadjuvant nivolumab + chemo	37	24	83	29.5	83	14	Stages IIIA–B
**AEGEAN**	Neoadjuvant durvalumab + chemo	33.3	17.2	77.6	39.6	94.7	13	Stages IIIA–B
**CHECKMATE-77T**	Neoadjuvant nivolumab + chemo	35.4	N/A	78	N/A	89	10	Stages IIIA–B
**NEOTORCH**	Toripalimab + chemo	48.5	N/A	82.2	N/A	95.8	16	Stages IIIA–B

## Data Availability

No new data were created or analysed in this study. Data sharing is not applicable to this article.
